# Scarifying the Gums

**Published:** 1869-11

**Authors:** 


SCARIFYING THE GUMS.—Dr. F. W. Hatch concludes an article on this subject in the California *Medical Gazette*, in these words:

1st. That while scarifying the gums in infancy is frequently of the most essential service in allaying nervous irritability, and relieving or warding off the early indications of cerebral congestion, the operation is not without danger, and like all others upon young children, should be performed only for just and sufficient causes.

2d. That it should be resorted to only on the most urgent indications, in systems already reduced by previous disease —pale, anæmic, and under the influence of mercury. That under these circumstances, it is better to risk the excitement due to difficult dentition than to resort to a remedy which may result fatally; in other words to bear and counteract the ills we have to deal with, than encourage others we know not of.

3d. That in the case referred to, the subject of this paper, there was an hemorrhagic diathesis, probably acquired as one of the effects of mercurial influence.

4th. That the condition of the system, especially of the blood, in similar cases, is such, that they must almost of necessity prove fatal.

